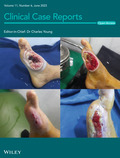# Cover Image

**DOI:** 10.1002/ccr3.7593

**Published:** 2023-06-19

**Authors:** Reham Alwis, Razan Alwis, Marwan Al‐Raeei

## Abstract

The cover image is based on the Case Image *Nanoparticles zinc paste bandages for the treatment of Syrian woman diabetic patient with ulcers in the foot: Case images* by Reham Alwis et al., https://doi.org/10.1002/ccr3.7445